# Baseline stool TIMP-2 predicts strictures and penetrating disease progression in Crohn’s patients

**DOI:** 10.3389/fimmu.2026.1624045

**Published:** 2026-04-10

**Authors:** Harshavardhan Polamarasetty, Ryan Pereira, Vinaika Maruvada, Kamala Vanarsa, Diptish Wankhade, Rajesh Yadavalli, Subra Kugathasan, Chandra Mohan

**Affiliations:** 1 Department of Biomedical Engineering, University of Houston, Houston, TX, United States; 2 Department of Pediatrics, Emory University, Atlanta, GA, United States

**Keywords:** Biomarkers, fecal proteomics, fecal TIMP-2, inflammatory bowel disease, pediatric Crohn’s disease

## Abstract

**Objective:**

Longstanding subclinical Crohn’s Disease (CD) can progress to complications such as intestinal strictures and penetrating (intestinal fistulae, abscesses, or perforations) disease. Despite the advances in non-invasive diagnostics for IBD, there is a dearth of biomarkers for predicting disease progression and complications in CD. Here, we investigate four baseline stool biomarkers, BDNF, MMP-8, TIMP-2, and Calprotectin (S100A8/A9), for their ability to predict disease progression in pediatric CD.

**Methods:**

Baseline stool samples from forty-seven patients recruited into the Pediatric RISK Stratification study were analyzed using an aptamer-based screening assay followed by validation by Enzyme-linked Immunosorbent Assays.

**Results:**

All four stool proteins were associated with disease activity at baseline. Baseline stool BDNF, MMP-8, and TIMP-2, but not Calprotectin distinguished progressors from non-progressors. Stool TIMP-2 predicted progression to stricturing and penetrating disease in CD, with higher levels predicting worse progression whereas an inverse relationship was seen with stool MMP-8, supporting the role of TIMP-2/MMP balance in driving fibrotic disease in CD. Indeed, stool TIMP-2 exhibited outstanding odds ratios of ~42 in predicting disease progression, outperforming all current protein or genetic markers of IBD. Stool BDNF was the next strongest performer associated with disease progression in CD.

**Conclusion:**

Stool TIMP-2 emerges as a novel predictor of disease progression in CD.

## Introduction

### Background

Crohn’s Disease (CD) is a multifactorial immune disorder characterized by chronic relapsing inflammation of the intestine (1). Over 20% of cases are diagnosed before the age of 17 making inflammatory bowel disease (IBD) one of the most common gastrointestinal chronic diseases affecting the pediatric population. The incidence of CD in children is increasing with an incidence of 2.5 to 11.4 per 100,000 and a prevalence of 58 per 100,000 (2). Along with Genetics, environmental factors, immune reaction against intestinal microbiota and gut dysbiosis have been shown as main triggers (3). Gut barrier dysfunction increases mucosal permeability to microbial antigens, and thus amplifies the inflammatory response in IBD (4). Immune dysregulation is a key player in the pathogenesis of IBD with abnormal dysregulation of T-cell subsets and hypersecretion of proinflammatory cytokines and subsequent immune response (5).

A definitive diagnosis often requires endoscopy in the management of CD. Several non-invasive stool biomarkers have been studied to assess their clinical utility in predicting clinical course and disease outcomes (6). Fecal calprotectin (S100A8/S100A9) is a widely used inflammatory marker, and its levels have been shown to correlate with endoscopic and histopathological metrics of disease activity and thus used as a reference standard (7). Longstanding subclinical disease activity can progress to complications such as stricturing (intestinal stenosis) and penetrating (intestinal fistulae, abscesses, or perforations) disease. These complications are present in 30%–50% of patients at diagnosis and occur in over 70% eventually (8). Currently, there are no established biomarkers that can predict whether or not a CD patient will eventually develop strictures or fistulas during follow-up. This study is designed to address this gap in our knowledge, by focusing on 4 specific stool proteins, namely BDNF, MMP-8, TIMP-2, and Calprotectin.

These 4 proteins were selected for study based on our previously completed stool biomarker screens in pediatric IBD patients (6, 9), together with the known functional properties of these proteins in IBD biology. In particular, Tissue inhibitors of metalloproteinases 2 (TIMP-2) and Matrix metalloproteinase 8 (MMP-8) exhibit a strong positive correlation with disease activity in IBD (6, 10). In addition, neurotrophic factors like Brain-derived Neurotrophic Factor (BDNF) expression in the intestinal mucosa has been reported to be increased in chronic IBD (11). Calprotectin is used as established standard for the diagnosis of IBD, and was hence included.

## Methods

### Human samples

The Pediatric RISK stratification study comprises pediatric Crohn’s Disease patients recruited from 28 centers in North America (12). Over 1800 CD patients have been prospectively recruited in this study with the collection of well-documented clinical, demographic, and biological samples every six months over three years, with continuing follow-up for five years (12). **Table 1** lists the demographic and clinical features of pediatric CD patients examined in our analyses, selected randomly from the RISK cohort. Inclusion criteria included patients younger than 18 years of age with endoscopy confirmed Inflammatory bowel disease. The exclusion criteria encompassed patients with diagnoses of inflammatory bowel disease other than Crohn’s Disease, patients with incomplete information on disease location, and those who experienced complications either at or within 90 days of diagnosis. Stool samples were obtained from these recruited patients at baseline through the IBD Plexus program and the Crohn’s & Colitis Foundation.

**Table 1:** Demographic and clinical information of pediatric Crohn’s disease subjects in different disease progression categories used in aptamer based proteomic assay.

Variable	Category	B1→B1	B1→B2	B1→B3	B1→B2+B3	Total
		(n=12)	(n=12)	(n=12)	(n=11)	(n=47)
Gender	Male	6	6	5	6	23
	Female	6	6	7	5	24
Race	White	11	1	3	0	15
	Not listed	1	11	9	11	32
wPCDAI^*^	Remission	5	0	2	0	7
	Mild	7	0	1	4	12
	Moderate	0	6	2	0	8
	Severe	0	6	5	6	17
	Not listed	0	0	2	1	3

•*wPCDAI, Pediatric Crohn’s Disease Activity Index.

•Phenotypes B1 = Inflammatory, B2 = Strictures, B3 = Fistulas, B2+B3 = Both strictures and fistulas.

Based on the distribution of stool biomarker levels in IBD and healthy controls observed for the top ten leading biomarkers, we calculated that a sample size of 12 per group will yield 80% power to detect differences at the 0.05 level of confidence (6, 9). Moreover, similar numbers have been sufficiently powered in our previous IBD stool biomarker studies (6, 9). All patients remained free from disease complications for the first 90 days of diagnosis and were subsequently followed at regular intervals. The clinical progression of their Montreal phenotype to Inflammatory (B1), stricturing (B2), penetrating (B3), or both stricturing and penetrating disease (B2+B3) over a 3–5 year follow-up period was documented (8). Additionally, the Weighted Pediatric Crohn’s Disease Activity Index (wPCDAI) was recorded at the time of enrollment to document the severity of the disease at baseline.

### Stool extraction

A total of 47 baseline stool samples from the RISK cohort, all at B1 phase at baseline, were used for proteomic and ELISA testing. The stool samples were weighed, added to the extraction buffer (NP lysis buffer and Protease Inhibitor), and subjected to 10 cycles of 2-minute vortex and 1-minute ice bath, following protocols we have previously reported (6). Subsequently, the samples were subjected to two rounds of centrifugation, and the clear supernatant was extracted. The final supernatant fraction was assayed for protein concentration using the BCA assay, aliquoted, and stored at -80 °C to maintain protein integrity for experimental use.

### Assay platforms used

We have used two biomarker detection methods – an aptamer based proteomic assay and ELISA, to measure baseline stool levels of the 4 candidate biomarkers TIMP-2, MMP-8, BDNF and S100A8/S100A9 and assessed their correlations with disease status. The aptamer-based assay is a molecular detection method that uses aptamers, a class of oligonucleotide probes which have the ability to bind to selective targets (8). Aptamers possess high affinity and specificity for their targets due to their capability of conformational binding to their target which could be small molecules (kd in picomolar range) or large proteins and even cells (13). In contrast to ELISA assays that use antibodies as baits, DNA-based aptamers are usually highly chemically stable (13). Indeed, a significant number of aptamer based screened identified biomarkers in our previous screens have been successfully validated by ELISA, as supported by our studies in lupus nephritis, IBD and various cancers (13–16).

### Aptamer based proteomic assay

The extracted stool samples were diluted to 20 µg/ml and subjected to an aptamer-based proteomic assay using validated aptamers (Somalogic Inc., Boulder, CO, USA). The current study focuses on four proteins namely TIMP-2, MMP-8, S100A8/A9, and BDNF included in this assay, based on previous studies reporting their diagnostic utility in IBD (6, 9). For the proteomic screen, we followed the protocol that has been reported previously (6). Briefly, stool samples were processed to bind proteins to aptamer-coated beads, washed to remove unbound proteins, and then biotinylated. After UV photocleavage, the proteins were dissociated, recaptured on streptavidin-coated beads, washed, and finally hybridized onto a DNA microarray to measure protein concentrations (6). The current report focuses on the 4 biomarker candidates selected based on previous literature, namely, TIMP-2, MMP-8, S100A8/A9, and BDNF. The rest of the proteins assayed in the proteomic screen are being analyzed further.

### ELISA assays

As an alternative assay method, TIMP-2, MMP-8, S100A8/A9, and BDNF were also assayed by ELISA (R&D systems) in the RISK cohort samples that were used for the aptamer-based assay. Optimal dilutions were first ascertained, and the ELISA assays were executed following the manufacturer’s instructions. The sample dilutions eventually used were 1:20 (TIMP-2), 1:50 (MMP-8), 1:100 (S100A8/S100A9), and 1:10 (BDNF) of the stool lysate. The levels of the four stool biomarkers were measured using standard curves generated by non-linear regression using Biotek Gen 5 software in Agilent Biotek microplate reader. The absolute levels of stool biomarkers were reported by normalizing the data to stool weight.

### Data analysis

Python and Microsoft Excel were used to plot and analyze the biomarker data. Subject groups were compared using the Mann–Whitney U test, following which p-values and q-values were calculated. Python was used to analyze significant differences between the groups tested. Outlier normalization was applied to minimize skew from extreme values, using the Interquartile Range (IQR) method for outlier detection. Outliers (defined as biomarker levels of samples lying below the 25^th^ percentile (Q1 – 1.5 x IQR) or above the 75^th^ percentile (Q3 + 1.5 x IQR) of the Inter quartile range (IQR) for more than 50% of the targets assayed) were median-normalized to the group median. This technique is implemented for correction of technical artifacts preserving biological signals (17, 18). Logistic regression, a supervised machine learning algorithm used for binary classification by estimating probabilities using a sigmoid function, was applied, to predict the odds of progression from B1 disease to B2 and/or B3 disease, using R.

## Results

**Table 1** lists the demographic and clinical features of the RISK CD patients examined. Of the 47 subjects from the RISK cohort used for the aptamer-based assay, their disease progression was as follows: 12 patients remained in group B1→B1 (non-progressors), 12 patients progressed from B1→B2 (progressors to B2), 12 patients progressed from B1→B3 (progressors to B3), and 11 patients progressed from B1→B2+B3 (progressors to B2+B3) over a 3–5 year follow-up period. Among them, the disease severity at baseline based on the wPCDAI was as follows: Remission = 7, Mild = 12, Moderate disease = 8, and Severe disease = 17.

**Figure 1:**
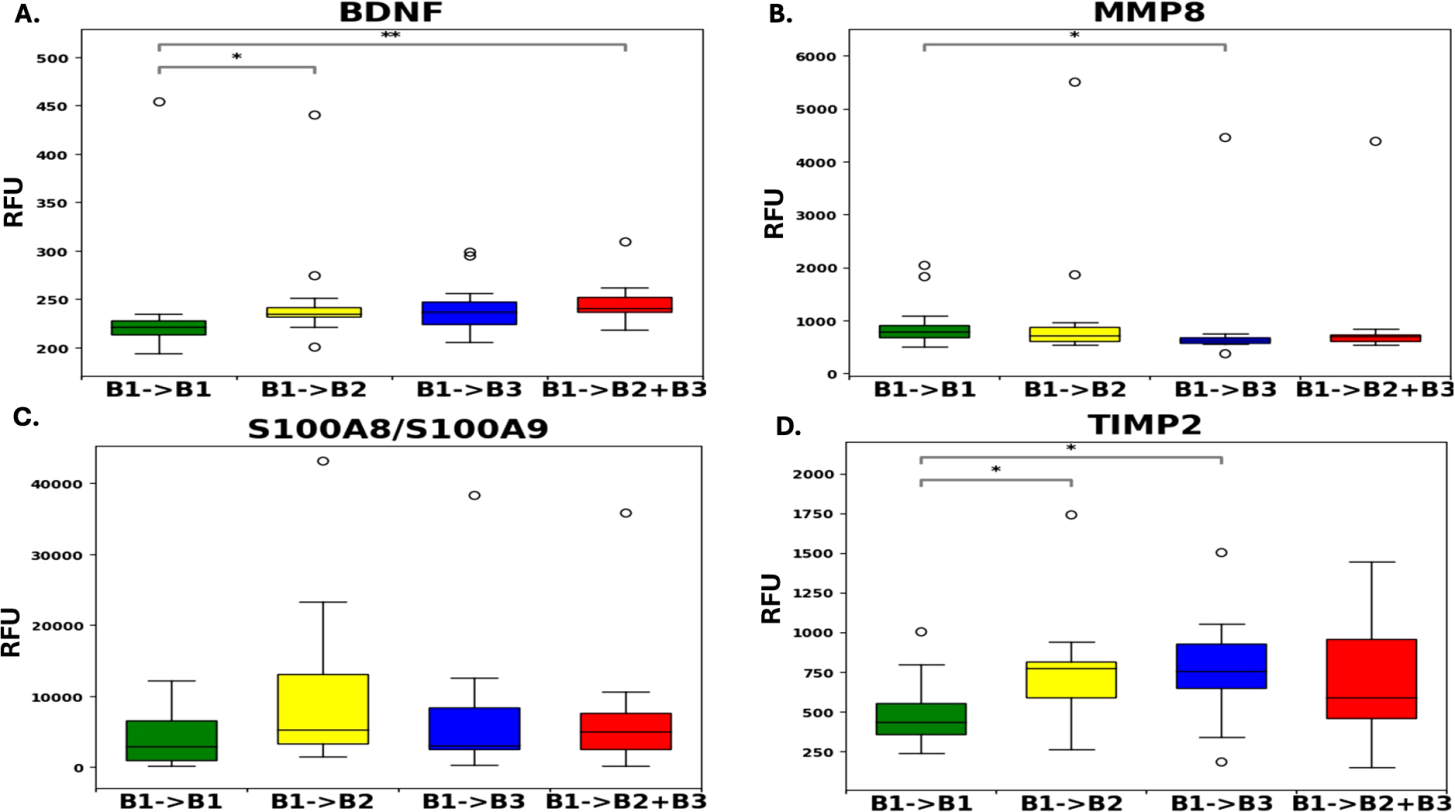
Distribution levels of four biomarker proteins **(A–D)** in pediatric Crohn’s disease stool as assayed by the aptamer-based assay. Plotted are the levels of four biomarker proteins in Pediatric Crohn’s disease stool as assayed by the aptamer-based screen using the RISK cohort of 12 B1→B1 samples, 12 B1→B2 samples, 12 B1→B3 samples and 11 B1→B2+B3 samples. *p<0.05, **p<0.01 based on a two-sided Mann-Whitney U-test conducted using Python. RFU – Relative fluorescent units; BDNF, Brain-derived Neurotrophic factor; MMP-8, Matrix metalloproteinase 8; S100A8/S100A9, Fecal calprotectin; TIMP-2, Tissue inhibitor of matrix metalloproteinases 2.

Results from the aptamer-based proteomic assay of the 4 candidate biomarkers examined showed that baseline levels of TIMP-2 were significantly higher in both progressors to B2 and progressors to B3 phenotypes compared to the non-progressors [p-value <0.05, fold change >1.5]. In contrast, stool levels of MMP-8 were significantly lower in progressors to B3 compared to non-progressors (p-value<0.05, fold change <1). Additionally, a significant increase in baseline stool BDNF levels was observed among progressors to B2 (p-value < 0.05, fold change > 1), and progressors to B2+B3 (p-value < 0.01, fold change >1) compared to non-progressors (**Figure 1**). Interestingly, baseline stool levels of both TIMP-2 and BDNF progressively increased from non-progressors to progressors to B2, and then to B3, while an inverse trend was noted with baseline stool MMP-8 levels. No significant changes were observed with fecal calprotectin.

**Figure 2:**
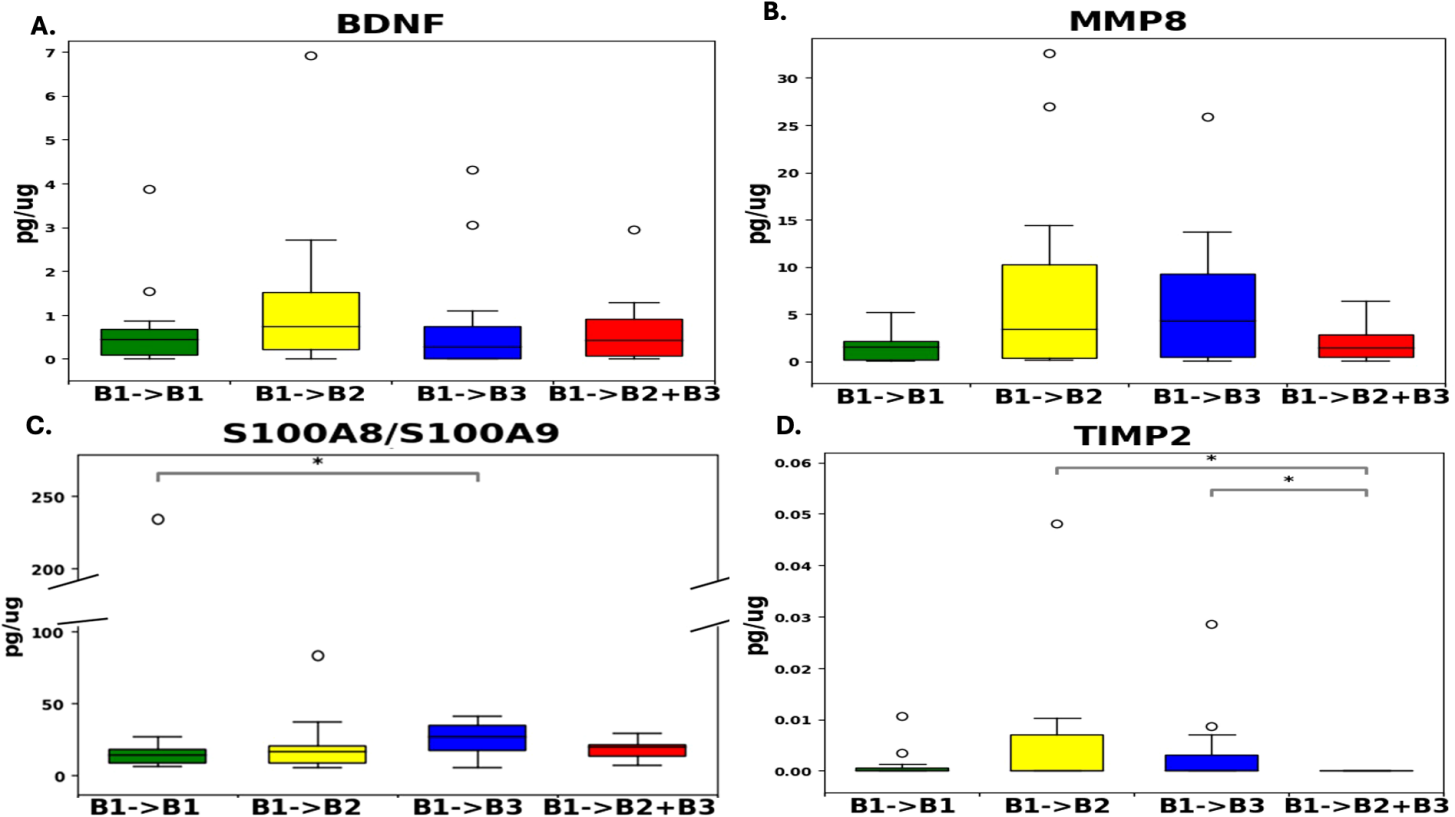
Distribution levels of the four biomarker proteins **(A–D)** at baseline in pediatric Crohn’s disease stool as assayed by ELISA. Plotted are the levels of four biomarker proteins in Pediatric Crohn’s disease stool as assayed by ELISA at baseline using the RISK cohort of 11 B1→B1 samples, 12 B1→B2 samples, 12 B1→B3 samples, and 8 B1→B2+B3 samples. *p<0.05, **p<0.01 based on a two-sided Mann-Whitney U-test conducted using Python. BDNF, Brain-derived Neurotrophic factor; MMP-8, Matrix metalloproteinase 8; S100A8/S100A9, Fecal calprotectin; TIMP-2, Tissue inhibitor of matrix metalloproteinases 2.

For the ELISA assay, a total of 43 RISK samples that were previously used for the aptamer-based proteomic assay were used, and among them, disease progression was as follows; non-progressors (n=11), progressors to B2 (n=12), progressors to B3 (n=12), and progressors to B2+B3 (n=8). Based on the ELISA assay, no statistically significant differences were found between the groups except for increased levels of S100A8/S100A9 (Calprotectin) between the non-progressors and progressors to B3 (p-value < 0.05) (**Figure 2**).

**Figure 3:**
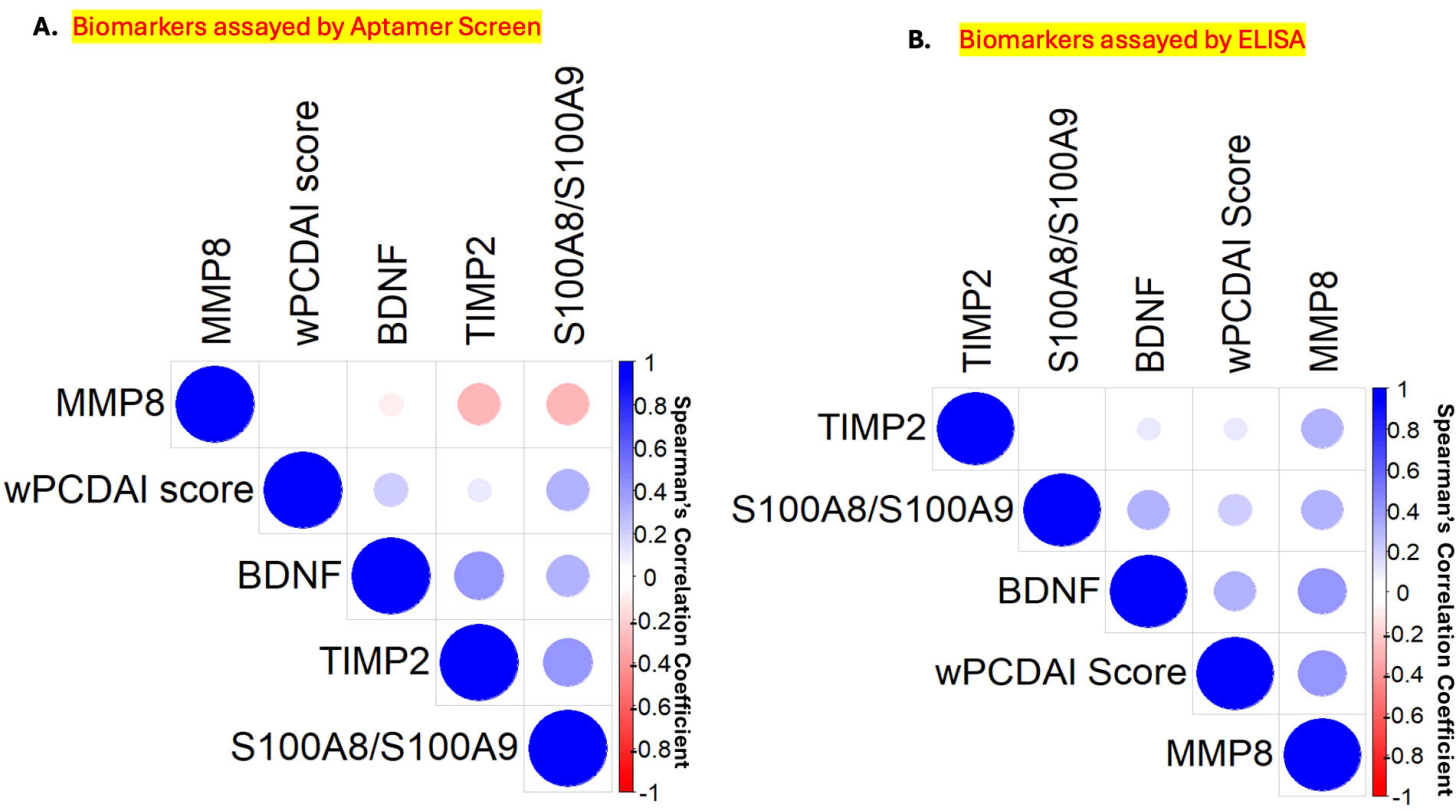
Correlation plot of stool biomarker levels and baseline weighted pediatric Crohn’s disease activity index analyzed by aptamer based assay **(A)** and ELISA validation **(B)**. **(A)** Spearman correlation analysis was performed between baseline stool levels of MMP-8, TIMP-2, S100A8/S100A9, and BDNF as assayed using the aptamer-based screen and baseline wPCDAI scores in 47 stool samples from the Pediatric RISK cohort. A positive correlation was observed between fecal S100A8/S100A9, TIMP-2, and BDNF and baseline wPCDAI scores. **(B)** Spearman correlation analysis was performed between the stool levels of MMP-8, TIMP-2, S100A8/S100A9, and BDNF as assayed by ELISA validation and baseline wPCDAI scores in 43 stool samples from the Pediatric RISK cohort. A positive correlation was observed among all the stool proteins and baseline wPCDAI scores. BDNF, Brain-derived Neurotrophic factor; MMP-8, Matrix metalloproteinase 8; S100A8/S100A9, Fecal calprotectin; TIMP-2, Tissue inhibitor of matrix metalloproteinases 2; wPCDAI, Weighted Pediatric Crohn’s Disease Activity Index.

Next, we examined if stool levels of MMP-8, TIMP-2, BDNF, and S100A8/S100A9, correlated with baseline disease activity. Aptamer-based analysis revealed a positive correlation between baseline wPCDAI scores and stool levels of S100A8/S100A9, TIMP-2, and BDNF but not with MMP-8 (**Figure 3A**). Whereas stool S100A8/S100A9, TIMP-2, and BDNF correlated with each other, MMP-8 did not. Similar correlation patterns were noted when the same stool proteins were assayed by ELISA (**Figure 3B**).

**Figure 4:**
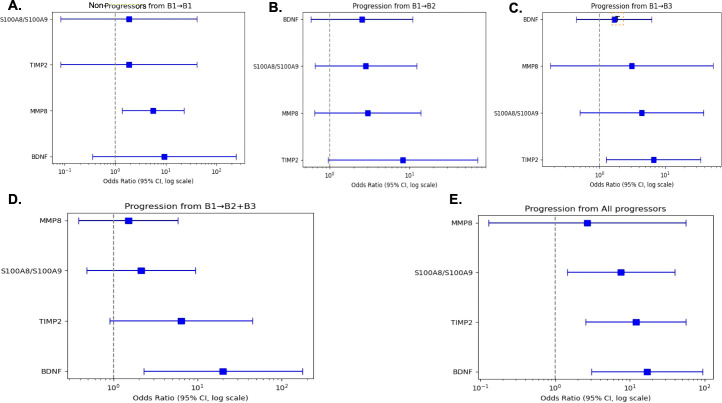
Forest plot displaying the odds of baseline stool biomarkers predicting progression to disease complications in CD. A logistic regression was performed between baseline stool levels of MMP-8, TIMP-2, S100A8/S100A9, and BDNF to predict the odds of progression from B1 to B1 **(A)**, B1 to B2 **(B)**, B1 to B3 **(C)**, B1 to B2+B3 **(D)**, and B1 to progressors **(E)** as assayed using the aptamer-based screen in 47 stool samples from the Pediatric RISK cohort. The forest plot displays the baseline stool TIMP-2 showed increased odds of Non-progressors from B1 to B1, B1 to B2 (OR 4.63, 95% CI 1.03-20.86), B1 to B3 (OR 3.33, 95% CI 1.05-10.55), and B1 to progressors (OR 41.95, 95% CI 1.41-1244.28). B1, Inflammatory; B2, Strictures; B3, Fistulas; B2+B3, both strictures and fistulas; progressors, B2, B3, and B2+B3; BDNF, Brain-derived Neurotrophic factor; MMP-8, Matrix metalloproteinase 8; S100A8/S100A9, Fecal calprotectin; TIMP-2, Tissue inhibitor of matrix metalloproteinases 2.

Additionally, we conducted a logistic regression analysis using the baseline stool levels of MMP-8, TIMP-2, S100A8/A9, and BDNF from aptamer-based analysis to calculate the odds of progression from B1 to B2, B1 to B3, B1 to B2+B3, or B1 to progressors (**Figure 4**). Interestingly, stool TIMP-2 showed increased odds of progression from B1 to B2 (OR 4.63, 95% CI 1.03-20.86), B1 to B3 (OR 3.33, 95% CI 1.05-10.55), and B1 to progressors (OR 41.95, 95% CI 1.41-1244.28). In contrast, the other baseline biomarkers failed to show significant odds of predicting disease progression.

## Discussion

Crohn’s Disease has a chronic relapsing and remitting disease course that requires frequent monitoring (6). Currently, endoscopy remains the major diagnostic tool in assessing mucosal healing as a therapeutic goal (19). Achieving Mucosal Healing (MH) is considered the main therapeutic goal by the International Organization of Chronic Inflammatory Bowel Diseases and the European Crohn Colitis Organization (20). Several studies have reported endoscopic scores defining MH and a recent study suggested optimal cut-off values for Modified Multiplier of the Simple Endoscopic Score -Crohn’s Disease (MM SES-CD) and Simple Endoscopic Score – Crohn’s disease (SES-CD) in predicting disease progression over a median follow-up of 3 years (21). Serum biomarkers like anti- *Saccharomyces cerevisiae* (ASCA) IgG and IgA antibodies, anti-CBir1(anti-flagellin), and anti-colony stimulating factor 2 (anti-CSF2) have been associated with stricturing disease phenotype in CD (22). A study reporting the association between higher serum levels of Collagen type III alpha chain 1 (COL3A1) in CD patients who developed strictures compared to non-progressors, is one such study that investigated the role of serum biomarkers in disease progression (22). However, there is a lack of similar studies investigating the utility of stool biomarkers in the disease progression of CD.

Fecal calprotectin or S100A8/S100A9 is the most widely used clinical inflammatory marker in IBD. It is a marker of neutrophil migration and has a better sensitivity than other inflammatory markers like C-reactive protein (6). Its levels also correlate with endoscopic and histopathological metrics of disease activity (6). Previous studies have reported that increased levels of fecal calprotectin were associated with progression in Montreal luminal behavior, surgical resection rates, and hospitalizations when studied in a retrospective cohort in CD (23). Another study showed that normalization of fecal calprotectin within the first 12 months of diagnosis of CD predicted a significantly lower risk of disease progression (24). However, in our study, fecal calprotectin failed to show a significant difference between the non-progressors and progressors in pediatric CD, as assayed by the aptamer screen or by ELISA.

Matrix metalloproteinases (MMPs) are a family of zinc-dependent peptidases that can degrade components of the extracellular matrix, including collagen. These enzymes are secreted as inactive zymogens by various cell types, including T-cells, macrophages, myofibroblasts, monocytes, neutrophils, and epithelial cells, and are subsequently activated in the extracellular space. Previous studies have shown both fecal and tissue expression of MMP-8 to be upregulated in IBD compared to controls (6, 10). Tissue Inhibitors of Matrix Metalloproteinases (TIMPs) form reversible complexes with MMPs regulating the extracellular matrix remodeling. Previous studies have shown higher expression of fecal TIMP-2 in IBD compared to healthy controls (6). Furthermore, longitudinal studies have shown a strong positive correlation of fecal MMP-8 and TIMP-2 with UC disease activity indices like Pediatric Ulcerative Colitis Activity Index (PUCAI) and Physicians Global Assessment (PGA) scores across different time points (6).

Interestingly, in the present study, whereas stool TIMP-2 progressively increased with disease progression in CD, stool MMP-8 exhibited an inverse pattern, based on the aptamer-based screen. This is consistent with the idea that increasing TIMP-2 may regulate the expression of MMP-8, consistent with the known biology of these proteins in tissue remodeling. It is known that an imbalance in MMP-TIMP activity leads to increased deposition of the extracellular matrix, facilitated by TIMPs, which shifts the balance away from MMPs, thus promoting intestinal fibrosis (25). Overexpression of TIMP-2 and its association with fibrosis is well documented in several organs including the liver, lungs, oral submucosa, heart, and brain (26–31). Overexpression of TIMP-2 has been reported to reduce the activity of MMP-8, -3, and -9 in lipopolysaccharide-stimulated mouse microglia (31). Similarly, the activity of MMPs is lower in Crohn’s disease who are more prone to fibrosis compared to UC patients (32). Based on these findings, we hypothesize that dysregulated TIMP/MMP balance, specifically the increase in TIMP-2 and a reciprocal decrease in MMP-8 may be a key promoter of stricture formation in CD.

In our study, baseline stool TIMP-2 levels exhibited significantly higher odds of predicting disease progression to stricturing and fistulizing complications, outperforming other contemporary markers in the published domain, including Single Nucleotide Polymorphisms (SNPs) in NOD2, IL-12B, TNFSF15, CX3CR1, IL-10, fecal calprotectin, and ASCA autoantibodies (22, 33–35). These studies strongly support the use of fecal TIMP-2 for risk stratification of patients diagnosed with IBD.

Brain-derived neurotrophic factor (BDNF) is a member of the neurotrophins family with various neuromodulatory effects in the CNS, gastrointestinal system, and pulmonary smooth muscle cells (5). Previous studies have highlighted its role in the pathogenesis of gastrointestinal disorders like IBD, Irritable Bowel Syndrome (IBS), and diabetic gastroenteropathy (11). Increased intestinal mucosal expression of BDNF is seen in chronic IBD, while polymorphisms in BDNF are associated with IBD susceptibility and severity (11). In the present study, the proteomic screen revealed a significant increase in stool BDNF among progressors to stricturing disease and progressors to both stricturing and penetrating disease compared to non-progressors. Interestingly, BDNF has been implicated in fibrosis in tissues like the lungs and liver (36–38). A recent study suggested that BDNF secreted from airway smooth muscle cells may increase extracellular matrix deposition, thus promoting fibrosis in asthma (37). Similarly, the activation of tyrosine kinase receptor B by the ligand BDNF, promoted epithelial-mesenchymal transition (EMT) in lung fibroblasts in Idiopathic Pulmonary Fibrosis (IPF) (39). These findings add to the growing evidence implicating BDNF as a mediator of fibrosis and underscoring its potential to serve as a marker of fibrotic complications.

Interestingly however, changes noted in the aptamer-based screen were not always concordant with the ELISA results. There could be several reasons for this. The aptamer-based proteomic screen uses specific deoxyoligonucleotides that bind with high affinity to target proteins with higher sensitivity (40). They can detect very low abundant proteins with greater sensitivity compared to immunoassays (41). ELISA, in contrast, uses antibodies as ligands for the target proteins. Hence, the epitopes interrogated by the two platforms may be different. Protein levels below the ELISA assay’s detection limit may indeed be responsible for the observed differences between the two platforms (41). We certainly have to entertain the possibility that the aptamer-based screen is yielding false positives, as has been suggested by others (42). Given these nuances, these findings need to be further expanded and validated. One may also need to perform ligand independent assays such as Mass Spectrometry, to confirm these findings.

To sum, our study provides evidence of baseline stool biomarkers associated with the progression of CD to stricturing and fistulizing complications. TIMP-2 and BDNF hold promise as they were significantly upregulated in the baseline stool of CD patients who later developed strictures and fistulas. These findings resonate well with the literature on these two proteins substantiating their role in promoting fibrosis in various organ systems. Furthermore, an inverse relationship was noted with stool MMP-8 in progression to stricturing complications supporting the known yin-yang balance of TIMPs/MMPs in promoting tissue remodeling and fibrosis. Indeed, baseline stool levels of TIMP-2 and BDNF but not MMP-8 correlate positively with baseline disease activity in CD.

From the clinical perspective, based on our findings, baseline stool TIMP-2 may serve as potential non-invasive tool for disease monitoring in CD replacing routine endoscopic surveillance and earlier identification of at-risk individuals. It could help decrease disease burden, procedural risks, waiting time and health care costs and help accelerate earlier identification of at-risk patients who are in disease progression. Earlier identification of at-risk individuals with CD may prompt more aggressive monitoring and treatment, leading to reduced morbidity, surgeries and hospital stays (43).

Future research will focus on (a) expanding these biomarker studies to larger prospective CD cohorts with more heterogenous patient populations, (b) expanding the follow-up interval for the longitudinal study, (c) studying both baseline and follow-up stool biomarker levels in the RISK CD cohort, (d) examining the diagnostic performance of the other proteins uncovered in our initial proteomic screen, (e) mechanistic studies to dissect out the pathogenic role of TIMP2/MMP8 balance in disease pathogenesis, and (f) to examine the therapeutic potential of targeting TIMP2/MMP8 in CD.

We acknowledge that several aspects of this study could be improved upon. Firstly, the sample size could be expanded to identify biomarkers with smaller effect sizes. Secondly, it is essential to expand our investigation through a comprehensive analysis of top stool proteins from the 7000-plex proteomic screen that might play a role in disease progression in CD. In addition, further longitudinal studies are warranted to investigate the true association of these biomarkers in patients with Crohn’s disease, over time, and their response to therapy. Finally, these studies need to be expanded to encompass adult CD patients and samples as well.

## Conclusion

In this study we found that baseline stool TIMP-2 is associated with disease progression from inflammatory disease to strictures/fistulas in Crohn’s disease. These findings highlight the potential of stool-based biomarkers to serve as noninvasive tests for early disease stratification, monitoring, and prognostication. If validated across larger prospective cohorts, these biomarkers may serve as new complementary tools replacing traditional clinical and endoscopic assessments for early diagnosis and disease monitoring of long-term complications of CD.

## Data Availability

The raw data supporting the conclusions of this article will be made available by the authors, without undue reservation.
